# Association of Sleep Problems With Dialysis Shifts in Patients Undergoing Hemodialysis in Tbilisi, Georgia

**DOI:** 10.7759/cureus.63733

**Published:** 2024-07-03

**Authors:** Nithesh Hariharan, Lasha Chkhikvadze, Ana Mateshvili, Ann Mary Sebastian, Naeba S Mathew, Elene Shavgulidze, Irma Tchokhonelidze

**Affiliations:** 1 Faculty of Medicine, American MD Program, Tbilisi State Medical University, Tbilisi, GEO; 2 Faculty of Medicine, American MD Program, Tbilisi State Medical University, Tbilsi, GEO; 3 Department of Nephrology, Tbilisi State Medical University and Ingorokva High Medical Technology University Clinic, Tbilisi, GEO

**Keywords:** chronic kidney disease (ckd), insomnia, poor quality sleep, sleep problems, dialysis shift, hemodialysis

## Abstract

Introduction

According to a 2023 poll by the International Society of Nephrology, 850 million individuals worldwide suffer from chronic kidney disease (CKD) and hemodialysis (HD) is the primary treatment for 69% of the patients with CKD. While HD effectively regulates fluid balance and electrolyte levels, patients often face challenges such as weakness, exhaustion, and cognitive changes, which impact their quality of life. Sleep-related issues, including poor quality, excessive morning sleepiness, insomnia, and restless leg syndrome (RLS), are particularly common among HD patients. These disturbances stem from various factors, including psychological discomfort and biochemical imbalances. Dialysis shifts, despite their importance, remain poorly studied regarding their impact on sleep and biochemical parameters. Our study aims to address these gaps, exploring how different dialysis shifts affect sleep quality and biochemical parameters. Our hypothesis suggests that the particular dialysis shift that hemodialysis patients undergo has an impact on the quality of sleep, with various groups exhibiting varying degrees of sleep disturbance. Simultaneously, we believe that the time of dialysis shifts could influence biochemical parameter variations, which in turn could affect the quality of sleep in hemodialysis patients.

Methodology

This cross-sectional study focuses on assessing sleep problems and analyzing biochemical variables among hemodialysis (HD) patients in Georgia. A total of 150 participants were selected from morning, afternoon, and evening dialysis shifts, with strict inclusion criteria and exclusion criteria. Assessment procedures involved questionnaires on sleep quality, restless leg syndrome (RLS), daytime sleepiness, and severity of insomnia. Biochemical variables were obtained from the hospital records. Statistical analyses were performed using Graph Pad Prism software (GraphPad, San Diego, USA), including ANOVA and Chi-square tests for association between biochemical variables and dialysis shifts, as well as logistic regression for assessing the influence of biochemical variables on insomnia and poor sleep quality. The significance level was set at 95%.

Results

Results showed that patients in the afternoon shift undergo longer sessions of hemodialysis compared to other shifts. Notably, a larger proportion of morning shift patients reported poor sleep quality, while a smaller fraction of evening shift patients experienced insomnia. There were no significant associations between dialysis shift and excessive morning sleepiness or restless leg syndrome. Potassium emerged as the sole biochemical variable exhibiting an association with all three dialysis shifts. Biochemical parameters showed no discernible impact on insomnia or poor sleep quality.

Conclusion

Our findings suggest an association between poor sleep quality and insomnia with dialysis shifts. Hemodialysis does influence potassium levels. However, biochemical variables like sodium, potassium, calcium, phosphorus, vitamin D3, parathyroid gland hormone (PTH), and hemoglobin do not seem to affect poor sleep quality and insomnia. Further research is needed to explore potential sleep issues with nocturnal shifts and to assess if creatinine and chloride have any influence on poor sleep quality. It is important to acknowledge dialysis shift as a contributor to sleep problems, emphasizing the need for targeted interventions to enhance the quality of life for these patients.

## Introduction

According to the International Society of Nephrology poll conducted in 2023, 850 million individuals worldwide were affected by chronic kidney disease (CKD) [1]. For 69% of this population, hemodialysis (HD) is the most common form of renal replacement therapy [2]. It assists in keeping the body's fluid and electrolyte balance in check by eliminating waste materials, and extra fluid, and controlling blood electrolyte levels. However, hemodialysis patients may experience complex issues like weakness, exhaustion, and changes in their emotional and cognitive abilities, all of which can have a major negative influence on their quality of life [3]. Among these difficulties, sleep-related problems are most prominent, which include poor sleep quality, excessive morning sleepiness, insomnia, and restless leg syndrome (RLS)[4].

The etiology of sleep disturbances in patients receiving hemodialysis is complex and involves a range of factors, from psychological factors such as discomfort related to hospital settings to biochemical contributors like elevated creatinine and urea, electrolyte imbalances, abnormal phosphate levels, imbalances of PTH, as well as anemia [5]. Among them, dialysis shifts, which refers to the scheduled times when patients receive hemodialysis, are a particularly understudied aspect of this problem. Typically structured into morning (5 AM - 10 AM), afternoon (10 AM - 3 PM), and evening (3 PM - 8 PM) shifts, each session varies in duration, frequency, and intensity of treatment [6].

Even though dialysis shifts are extremely important, not much study has been done on how they might affect hemodialysis patients and their sleep-related results. Studies that have already been conducted yield inconsistent results. While one study claims that patients in the morning shift exhibit better sleep quality compared to those in the evening shift [7], another study finds no evidence linking the dialysis shift to sleep issues [8]. Furthermore, important biochemical parameters in hemodialysis patients, such as potassium, sodium, calcium, bicarbonate, urea, phosphates, and hemoglobin may be impacted by dialysis shifts [9]. Patients on hemodialysis may experience sleep difficulties as a result of variations in certain biochemical parameters.

In addition, not much research has been done on how the dialysis shift affects sleep disorders. The limited amount of research that is currently available suggests a possible relationship between insomnia and morning-shift dialysis and prolonged life expectancy [9]. However, a comprehensive understanding of the comparative influence of dialysis shifts on clinical manifestations, particularly concerning excessive morning sleepiness and restless leg syndrome (RLS), remains to be clarified.

Sleep disturbances in dialysis patients have wide-ranging negative effects beyond the previously mentioned declines in quality of life. The long-term implications of sleep apnea on morbidity and mortality in dialysis patients remain unknown, but its link to cardiovascular disease, the primary cause of death in this population, highlights the importance of diagnosing and controlling sleep apnea for improved clinical outcomes [5]. Dialysis patients may experience autonomic dysfunction as a result of nocturnal hypoxemia [9]. Periodic limb movement disorder (PLMD) and restless legs syndrome (RLS) are also linked to higher death rates, which calls for more extensive long-term research on the impact of treatment on dialysis patients [7].

To bridge these knowledge gaps, our study aims to investigate whether sleep issues are present in hemodialysis patients during various dialysis shifts. In addition, we intend to study the impact of dialysis shifts on key biochemical parameters, postulating that modifications in these values may be a contributing factor to sleep-related issues in the hemodialysis patients. Additionally, we want to evaluate any possible connection between the frequency of sleep disturbances and the time of dialysis shifts.

## Materials and methods

Subjects

This research is a cross-sectional study based on questionnaires about sleep problems and analysis of biochemical variables of hemodialysis patients. This research study concentrates on the population that has been undergoing hemodialysis in Ingorokva Clinic (High Technology Medical Center, University Clinic), Tbilisi, Georgia. 50 patients were selected from Morning (5 AM - 10 AM), Afternoon (10 PM - 3 PM), and Evening (3 PM - 8 PM) dialysis shifts respectively, with a collective total of 150 participants for our study, which is a similar number of participants in other studies mentioned in the discussion. Participants were undergoing hemodialysis twice a week for the past 3 months at the same dialysis shift. Participants, including males and females, diagnosed with any stage of chronic kidney disease and of the age group, from 18 years to 80 years were strictly selected. This is because the intrinsic circadian timing system and the homeostatic sleep-wake system of those under the age of 18 are not yet matured which causes them to have an overall short sleep duration [10] and those above 80 years have difficulty falling asleep and maintaining sleep, early-morning awakening and excessive daytime sleepiness, etc. [11]

Patients with a history of sleep problems before initiation of hemodialysis such as insomnia, restless leg syndrome, etc., or a history of psychiatric disorders or any other cognitive disorder that affects sleep in patients such as anxiety, depression, pseudodementia, hypothyroidism, or use of any kind of stimulants like ramelteon, zolpidem, etc., were excluded from participants [12].

Assessment Procedure

The questionnaire included consent of the participant, name, age, height, weight, and duration of ongoing dialysis (in months). To assess different kinds of sleep problems, our study included an assessment of sleep quality by the Pittsburgh Sleep Quality Index (PSQI), an assessment of restless leg syndrome (RLS) by RLS criteria, an assessment of daytime sleepiness by Epworth Sleepiness Scale (ESS) and assessment of insomnia by Insomnia Severity Index (ISI). These standardized tests have been used in previous studies which have shown to effectively evaluate a participant's sleep problem and proved that they are ideal for diagnosing participants with sleep problems [13-16]. 

Pittsburgh Sleep Quality Index (PSQI) scale consists of seven components: a) subjective quality of sleep; b) sleep onset latency; c) sleep duration; d) sleep efficiency; e) presence of sleep disturbances; f) use of hypnotic-sedative medication; and g) presence of daytime disturbances, as an indication of daytime alertness. Participants having a score of 5 or more were considered to be positive for poor quality of sleep [13].

Restless Leg Syndrome (RLS) questionnaire included four aspects: a) an urge or unpleasant sensation in the legs (numbness, crawling of ants, burning, tingling, movement of worms, electric shock, kicking, etc.) associated with the impulse to move the legs; b) an unpleasant sensation or insatiable urge to move your legs only or mostly when you are at rest (eg: sitting or lying down); c) Movement, such as walking or stretching, relieves the want to move or unpleasant sensations, at least for the duration of the activity and d) if the urge to move the leg is more intense in the evening or night than in the morning. These four questions were to be answered as Yes or No, and if four out of four turned out to be true, the subject was considered to be positive for RLS [14].

Epworth Sleepiness Scale (ESS) contained eight hypothetical situations whose answers may vary from zero (no probability) to three (high probability). A score of 10 or more indicated Excessive daytime somnolence [15].

Insomnia Severity Index (ISI) is a seven-component questionnaire: a) falling asleep; b) staying asleep; c) early awakening; d) satisfaction; e) interference; f) noticeable; g) worry. Each component was measured from zero (no problem) to four (very severe). Total score of 8 or more qualified for Insomnia in the subject [16].

To guarantee that the participants understood the questions well, these four questionnaires were combined into one sleep questionnaire, translated into Georgian by a professional Georgian-English speaker, and then translated back into English by another professional Georgian-English speaker. This helped us understand if the English questions were appropriately translated into Georgian and if there was no mistranslation of the questionnaire during the process of translation. To check if the participants were able to understand this questionnaire, a pilot study was conducted with five participants from the morning, afternoon, and evening shifts. The participants took an average of 7 minutes (SD 2 minutes) and the participants did not have difficulty understanding the questionnaire.

Biochemical variables were taken from hospital records for each patient in different dialysis shifts that were most recently measured or measured within the last 6 months prior to meeting the participants. Laboratory values of Calcium (Ca 2+), Blood Urea Nitrogen (BUN), Phosphorus (P), Parathyroid Hormone (PTH), 25 (OH) Vitamin D3, Hemoglobin, Sodium (Na+ ) and Potassium (K+ ) were recorded.

Statistical Analysis

Statistical analysis for this research was done using GraphPad Prism Version 10.1.2 (324)]software (GraphPad, San Diego, USA). The association of biochemical variables with dialysis shift was done using ANOVA for quantitative analysis. Association of different sleep problems with dialysis shift was done using Chi-square for categorical data. Influence of biochemical variables on insomnia and poor quality of sleep was done using multiple logistic regression. Post hoc analysis was done in SPSS (IBM Corp., Armonk, USA). Patients were stratified according to dialysis shift and statistical significance was defined at a 95% level (p<0.05)

## Results

Demographical data

Over the course of many dialysis shifts, the research subjects showed diversified demographic patterns. As seen in Table 1, the morning shift had 35 male and 15 female participants. The afternoon shift had 18 males and 32 females and the evening shift had 29 males and 21 females. The mean age of the participants among the morning shift dialysis participants (Mean ± SD) was 59.82 ± 3.164, 63.28 ± 3.274 among the afternoon shift participants, and 58.2 ± 3.58 among the evening shift participants. The mean BMI (Mean ± SD) was found to be 25.70 ± 1.04 among the morning shift participants, 26.35 ± 1.59 among the afternoon shift participants, and 26.51 ± 1.84 among the evening shift participants. The mean duration of dialysis (Mean ± SD) for the morning, afternoon, and evening shifts were 68.86 ± 21.337, 94.02 ± 20.64, and 66.46 ± 16.9 minutes, respectively.

**Table 1:** Demographic characteristics of patients undergoing hemodialysis P-value obtained from analysis of variance (ANOVA)

MEAN ± SD	MORNING	AFTERNOON	EVENING	p-value
Age (Years)	59.82 ± 3.164	63.28 ± 3.274	58.2 ± 3.58	p=0.2
BMI (kg/m^2^)	25.70 ± 1.04	26.35 ± 1.59	26.51 ± 1.84	p=0.8
Duration of dialysis (months)	68.86 ± 21.337	94.02 ± 20.64	66.46 ± 16.9	p=0.04
Male/Female	35/15	18/32	29/21	p=0.4

Using ANOVA to assess if dialysis shift had any influence on the demographical data, it was found that the p-value for the mean age (0.2) and BMI (0.8) was more than 0.05, hence proving it to be statistically insignificant. On the other hand, doing analysis using ANOVA for the duration of dialysis and dialysis shift, the p-value was 0.04 proving that the duration of dialysis is influenced by the dialysis shifts. Post hoc analysis with Tukey's Kramer test was done to identify which shift has an influence on the duration of dialysis. The mean difference in duration of dialysis between the afternoon and morning shifts was 25.16 (p=0.04) and the mean difference in duration of dialysis between the afternoon and evening shifts was 27.56 (p=0.03). Both p-values are statistically significant, which means that the afternoon shift has an influence on the duration of dialysis. However, on comparing the mean duration of dialysis of the morning shift with the afternoon (p= 0.2) and evening shift (p=0.073), it was not statistically significant. Similarly, on comparing the mean duration of dialysis of the evening shift with the afternoon shift (p=0.20) and morning shift (p=0.81), it was not statistically significant. 

Sleep problems in each dialysis shift

As seen in Table 2, 36 participants from the morning shift, 24 participants from the afternoon shift, and 28 participants from the evening shift had poor-quality sleep. Using Chi-square to check if dialysis shift had any influence on poor sleep quality, the p-value was 0.04, proving that dialysis shift does have an influence on the poor quality of sleep. Post hoc analysis revealed that the adjusted p-value for the cell which compared poor quality of sleep with the morning shift is significant (p=0.0032). However, afternoon shift (p=0.6) and evening shift (p=0.075) did not have any significant influence on poor quality of sleep.

**Table 2:** Results of the Pittsburgh Sleep Quality Index (PSQI), Epworth sleepiness scale (ESS), Restless Leg Syndrome (RLS), and Insomnia Index in 150 patients undergoing hemodialysis in shifts. P-value obtained from Chi-Square test.

Sleep Problem	Morning Shift	Afternoon Shift	Evening Shift	P value
Poor Quality of Sleep	36	24	28	p=0.04
Excessive Morning Sleepiness	13	10	4	p=0.05
Restless Leg Syndrome	15	21	13	p=0.2
Insomnia	34	29	13	p=0.001

The Insomnia Index yielded a highly significant difference among the shifts (p = 0.001). Table 2 shows 34 participants from the morning shift with insomnia, followed by 29 in the afternoon and 16 in the evening shifts. Post hoc analysis revealed that the adjusted p-value for the cell comparing insomnia with evening shift is significant (p=0.0032). However, morning shift (p=0.08) and afternoon shift (p=0.556) did not have any significant influence on insomnia. The results proved that participants from the evening shift had a lesser chance of having insomnia in comparison to the morning and afternoon participants.

In Table 2, it is shown that 35 participants in the morning had restless leg syndrome (RLS), followed by 29 participants from the afternoon shift and 37 participants from the evening shift. The p-value (0.2) obtained after analyzing using Chi-square between RLS and dialysis shift was more than 0.05 proving it to be statistically insignificant in this study. The scores for the Epworth Sleepiness Scale (ESS), which assessed excessive morning sleepiness showed a trend, even though they fell short of statistical significance (p = 0.06). As shown in Table 2, 13 participants from the morning shift were seen to have more excessive morning sleepiness compared to 10 participants from the afternoon shift and four from the evening shift.

Biochemical variables in each dialysis shift

This study also examined the effects of various shifts on the various biochemical variables of the participants, as shown in Table 3. The potassium level in the morning shift (Mean ± SD) was 4.75 ± 0.16, 5.2 ± 0.17 in the afternoon shift, and 4.72 ± 0.21 in the evening shift. ANOVA showed that dialysis shift has an influence on potassium levels (p=0.04). Post hoc analysis with Tukey's Kramer test was done to identify which shift has an influence on potassium levels. On analysis, there was no significant difference in potassium levels between morning shift with afternoon (p=0.6) and evening shift (p=0.056). Similarly, there was no significant difference in potassium levels between the afternoon shift and the potassium levels of the morning (p=0.6) and evening shifts (p=0.07). Finally, there was no significant difference in potassium levels between the evening shift and the potassium levels of the morning (p=0.9) and afternoon shifts (p=0.97). The dialysis shift did not have any effect on the total calcium, blood urea nitrogen (BUN), and phosphorous levels, as their p-value was seen to be more than 0.1 proving them to be statistically insignificant in this study. There was no discernible change in the levels of vitamin D3 between shifts (p = 1) and no significant shift-related changes were seen in the levels of hemoglobin, sodium, or parathyroid hormone (PTH) (p=0.2).

**Table 3:** Mean and standard deviation of biochemical parameters in each dialysis shift P-value obtained from ANOVA.

MEAN	MORNING	AFTERNOON	EVENING	P-value
Potassium (mmol/L)	4.75 ±0.16	5.2 ±0.173	4.82 ±0.21	p=0.045
Total Calcium (mmol/L)	2.14 ±0.058	2.15 ±0.04	2.09 ±0.06	p=0.2
Blood Urea nitrogen (BUN) (mg/dL)	50.66 ±14.92	39.2082 ±6.78	40.2242 ±9.13	p=0.2
Phosphorus (mmol/L)	1.84 ±0.14	1.65 ±0.12	1.68±0.113	p=0.1
Vitamin D3 (nmol/L)	40.1737 ±6.939	41.29 ±8.88	37.65 ±6.51	p=1
Hemoglobin (g/dl)	10.60 ±0.42	10.94 ±0.36	11.08 ±0.41	p=0.2
sodium (mmol/L)	135.47 ±0.82	136.17 ±0.77	136.53 ±1.066	p=0.5
Parathyroid Hormone (pg/ml)	501.30 ±139.09	459.79 ±95.89	426.6166 ±94.93	p=0.4

Biochemical variables and sleep problems

Using multiple logistic regression analysis to check if any biochemical variables had any influence on poor quality of sleep and insomnia, it was found that age, duration of dialysis, potassium, calcium, blood urea nitrogen (BUN), phosphate, parathyroid hormone (PTH), vitamin D3, hemoglobin, and sodium did not show any association with participants who had PSQI more than 5 (poor quality of sleep) and those with PSQI less than 5. This analysis was carried out by grouping each parameter against participants with PSQI and with those without PSQI separately. As seen in Table 4, the p-value of age (0.29), duration of dialysis (0.43), potassium (0.99), calcium (0.22), BUN (0.34), phosphate (0.88), PTH (0.41), D3 (0.65), hemoglobin (0.41) and sodium (0.77) for each of it was found to be more than 0.05 proving them to be insignificant in this study.

**Table 4:** Mean and SD of various parameters in patients with insomnia and without insomnia. P-value obtained using multiple logistic regression.

Parameters	Insomnia (79)	No insomnia (71)	P value
Age (years)	61.8 ±2.30	59.20 ±4.10	0.29
Duration of dialysis (months)	41.13 ±12.51	49.79 ±19.52	0.43
Potassium (mmol/L)	4.83 ±0.17	4.94±0.32	0.99
Calcium (mmol/L)	3.8 ±0.32	2.11 ±0.08	0.22
Blood Urea Nitrogen (mg/dl)	39.54 ±10.02	37.0 ±8.70	0.34
Phosphate (mmol/L)	1.709 ±0.11	18.20 ±3.2	0.88
Parathyroid Hormone (pg/ml)	488.01 ±125.83	609.6 ±211.51	0.41
Vitamin D3 (nmol/L)	39.48 ±9.72	27.44 ±6.52	0.65
Hemoglobin (g/dl)	10.89 ±0.35	13.59 ±5.74	0.41
Sodium (mmol/L)	136.54 ±0.741	136.25 ±1.42	0.77

Similarly in the case of the insomnia index, as shown in Table 5, the p-value of age (0.24), duration of dialysis (0.44), potassium (0.99), calcium (0.22), BUN (0.28), phosphate (0.88), parathyroid hormone (0.41), vitamin D3 (0.65), hemoglobin (0.41), sodium (0.77) were all more than 0.05, proving that these parameters did not show any association with participants who had insomnia and those who did not have insomnia.

**Table 5:** Mean and SD of various parameters in patients with poor quality of sleep and normal quality of sleep. P-value obtained using multiple logistic regression.

Parameters	PSQI (88 participants)	Normal Sleep (62 participants	P value
Age (years)	62.18 ±6.52	59.78 ±3.92	0.24
Duration of dialysis (months)	46.17 ±12.33	28.63 ±16.67	0.44
Potassium (mmol/ml)	4.86 ±0.21	4.83 ±0.26	0.99
Calcium (mmol/ml)	3.25 ±0.32	2.12 ±0.06	0.22
Blood Urea Nitrogen (mg/dl)	46.71 ±14.04	46.31 ±10.72	0.28
Phosphorus (mmol/l)	9.97 ±16.34	1.97 ±0.18	0.88
Parathyroid Hormone (pg/ml)	428.35 ±80.92	576.68 ±27.03	0.411
Vitamin D3 (nmol/L)	42.11 ±9.49	29.23 ±6.35	0.650
Hemoglobin (g/dl)	12.432 ±2.87	10.65 ±0.57	0.412
Sodium (mmol/l)	136.92 ±0.78	136.11 ±1.28	0.779

## Discussion

Disruptions in sleep are a prevalent issue observed among individuals undergoing hemodialysis, and one of the contributing factors to sleep problems is the dialysis shifts. The presence of sleep problems in hemodialysis patients not only leads to a decline in their overall quality of life but also serves as a potential factor contributing to increased mortality within this patient population [17].

Firstly, our study revealed that patients in the afternoon shift had a longer duration of dialysis, compared to the other shifts. Also, our study revealed notable findings regarding the potential impact of dialysis shifts on sleep. Specifically, a higher number of patients in the morning shift exhibited poor quality of sleep, compared to the patients in the afternoon and evening shifts. Conversely, a lower number of patients in the evening shift had insomnia, compared to both morning and afternoon shifts. Despite the influence of dialysis shift on sleep quality and insomnia, our results indicated that restless leg syndrome (RLS) and excessive morning sleepiness were not influenced by dialysis shift. Upon analyzing the potential influence of dialysis shift on biochemical values, only potassium had a significant association. While there were differences in biochemical factors among individuals experiencing poor sleep quality compared to those without such issues, the lack of statistical significance suggests that these laboratory values do not significantly impact the quality of sleep. Similar results were observed in relation to insomnia.

Examining the influence of dialysis shift on demographic data revealed an intriguing finding, which was in contrast to that by Bastos et al. [17]. Our research indicated that the dialysis shift significantly affected the duration of dialysis, with individuals in the afternoon shift undergoing longer sessions compared to those in the morning and evening shifts. In contrast, Bastos et al. reported no influence of dialysis shift on the duration of dialysis [17]. One possible explanation for this disparity is that the afternoon shift in our study hospital started at 10 AM and concluded at 3 PM. Patients, who have been doing dialysis for a long period of time seemed to prefer this time, compared to that of morning and evening shifts. The afternoon shift allowed them to avoid early wake-ups, and they could finish their sessions by 3 PM, providing ample time for their daily activities and recovery from hemodialysis. Therefore, the observed impact on duration may not be a direct result of the dialysis shift but rather a preference among patients with longer hemodialysis histories for the convenient timing of the afternoon shift.

Wang et al. showed that patients undergoing hemodialysis during the morning shift had a better quality of sleep, compared to the afternoon and evening shifts [18]. This improvement was attributed to the active lifestyle as well as optimized biochemical clearance during this timeframe. On the other hand, Bastos et al. have shown that, although patients undergoing hemodialysis had different sleep problems, including poor quality of sleep and insomnia, there was no significant association with dialysis shift [17]. Contrary to previous research, our study indicated that patients in the morning shift have poor quality of sleep compared to those in the afternoon and evening shifts. The morning shift, occurring from 5 AM to 10 AM, necessitates patients to be at the hospital by 5 AM, disrupting their normal circadian rhythm. Research by Flaskerud et al. highlighted that people waking up early, like 3 AM, disrupts their natural physical and emotional recovery and hence, they are likely to have stress, anxiety as well as poor quality of sleep [19]. The hospital environment further contributes to sleep challenges for morning-shift patients. The atmosphere and sounds in the hemodialysis unit may make it uncomfortable for patients to sleep and even if they do, the quality of sleep is poor and compromised. Given the requirement for hemodialysis on alternative days, patients struggle to establish a consistent circadian rhythm, potentially exacerbating their already poor sleep quality. Consistent disruptions to the body's internal clock can contribute to insomnia by affecting the regularity of sleep patterns. The afternoon and evening shift occurs at 10 AM - 3 PM and 3 PM - 8 PM respectively, thereby not interfering with their normal circadian rhythm and hence, less likely to develop poor quality of sleep.

Similarly, findings from Bastos et al. showed that dialysis shift has no impact on insomnia [17]. In contrast, our study has shown that dialysis shifts have an influence on insomnia, specifically highlighting that patients in the evening shift have a lower prevalence of insomnia, compared to those in morning and afternoon shifts. Several factors contribute to this observation. Firstly, the evening shift, spanning from 3 PM to 8 PM, enables patients to avoid early wake-ups, distinguishing it from both morning and afternoon shifts. Additionally, evening shifts align more closely with the natural circadian rhythm of individuals, allowing for better synchronization with the body's internal clock. This alignment can reduce the likelihood of insomnia.

As highlighted by Hassen et al. it is very critical to understand the effect of hemodialysis, more specifically, dialysis shift, on routine biochemical variables [20]. Our research concentrated on biochemical variables that were routinely measured in the hospital, encompassing sodium, potassium, calcium, phosphorus, vitamin D3, PTH, and hemoglobin. Given that these biochemical variables influence the overall morbidity and mortality of patients, comprehending the potential impact of dialysis on these laboratory values is crucial. This understanding enables better supervision, leading to enhanced quality of life and reduced patient mortality [20,21]. Hassen et al. showed that hemodialysis has an influence on potassium and phosphorus, where patients undergoing hemodialysis had higher levels of phosphorus and potassium, compared to the control group [20]. Our study showed that dialysis shift has an influence on potassium. Post hoc analysis showed that no specific shift has influence on potassium. This could mean that dialysis shift has an influence on potassium in general, but no specific shift has influence on potassium. Although potassium was normal in all the dialysis shift groups, the fact that all three shifts had an influence is crucial in understanding that hemodialysis has an impact on potassium, which supports Hassen et al. [20]. Conversely, our findings indicate that dialysis shift had no influence on other biochemical variables such as calcium, phosphorus, PTH, vitamin D3, hemoglobin, and sodium, aligning with the conclusions from the study by Bastos et al. [17]

Numerous studies have explored the impact of biochemical factors on sleep patterns. Sabbatini M et al. findings indicated a significantly higher risk of insomnia in individuals with elevated parathyroid hormone (PTH) levels, while no difference in hemoglobin concentrations was observed between insomnia and non-insomnia groups [22]. Hejazian et al. reported a negative correlation between serum vitamin D levels and overall sleep quality, even after adjusting for confounding factors like calcium (Ca), phosphate (P), and PTH levels [23]. On the contrary, research by Iliescu et al. showed no statistically significant relationship between the global PSQI score and blood urea nitrogen (BUN) levels, as well as hemoglobin [24]. Our study found no significant influence of sodium, potassium, calcium, phosphorus, vitamin D3, PTH, and hemoglobin on insomnia. This contradicts the controversial findings. Our study aligns with Iliescu et al. findings, indicating that sodium, potassium, calcium, phosphorus, vitamin D3, PTH, and hemoglobin have no influence on poor sleep quality. Consequently, the observed insomnia and poor sleep quality in our study are more likely attributed to the timing of dialysis shifts rather than these biochemical variables.

Bastos et. al has shown that understanding the factors behind sleep problems in hemodialysis patients is crucial for their overall well-being and mortality of the patients, as well as for improving their health status [17]. As outlined in the National Kidney Dialysis and Kidney Transplantation Study, disruptions in sleep have been identified as significantly detrimental to functional health, as indicated by the Sickness Impact Profile [5]. Such sleep disturbances may lead to the onset or exacerbation of other concurrent health issues like hypertension and cardiovascular conditions, consequently elevating the mortality risk for hemodialysis patients. Additionally, disturbances in sleep among individuals undergoing hemodialysis can impact family interactions [5] and are linked to heightened levels of anxiety, depression, and increased days of disability [25]. Understanding all the potential reasons behind sleep problems in patients undergoing hemodialysis can help us find curative ways for these reasons and thereby, increase to improve the rehabilitative potential of hemodialysis.

Given that our study has indicated that dialysis shifts can influence poor sleep quality and insomnia, certain interventions can be done to help improve their sleep. Health workers can make individualized dialysis schedule that considers patient’s medical history, lifestyle, and sleep patterns to improve their quality of life. Hemodialysis centers can provide better times to schedule dialysis to minimize negative effects on patient’s sleep. Furthermore, in some countries (though not in Georgia), an additional hemodialysis shift, known as the nocturnal shift, is offered [26]. Nocturnal dialysis involves a slower and longer hemodialysis treatment conducted during the night while the patient sleeps. This extended treatment lasts for six to eight hours, occurring three times a week or more, as mentioned by the National Kidney Foundation. Wong et al.'s research suggests that nocturnal hemodialysis is associated with improvements in various clinically relevant outcomes, including enhanced systolic blood pressure, increased hemoglobin levels, and lower serum phosphate levels [26]. However, there is currently no study examining the impact of the nocturnal shift on sleep, highlighting the need for further research in this area.

Our study has certain limitations that warrant consideration. Firstly, we did not account for potential co-morbid conditions in our analysis. Lo K et al. highlighted that patients with hypertension are at an increased risk for poor sleep quality [27]. A cross-sectional study is not useful in understanding the causality of these sleep problems. Also, it is important to understand and obtain more information on secondary causes of RLS such as Parkinson's disease, peripheral neuropathy, diabetes mellitus, iron deficiency, etc., which can act as confounding factors [14]. Sleep itself is a self-reported evaluation, which can result in recall bias. As our study showed the association of dialysis with hyperkalemia, it is essential to consider the patient's chronic illness and medications, which may influence potassium levels [28]. Additionally, it is important to take other potential confounding factors that affect sleep quality into consideration, such as stress, adiposity, and socioeconomic status into consideration [29]. Other hemodialysis centers with slightly different shift timings could also contribute to variations.

## Conclusions

In summary, our findings suggest an association between poor sleep quality and insomnia with dialysis shifts. Hemodialysis does influence potassium levels. However, biochemical variables like sodium, potassium, calcium, phosphorus, vitamin D3, PTH, and hemoglobin do not seem to affect poor sleep quality and insomnia. Further research is needed to explore potential sleep issues with nocturnal shifts and to assess if creatinine and chloride have any influence on poor sleep quality. Future studies on this topic will help us have a better understanding of the causality of these sleep problems (by doing a longitudinal study). By understanding causality, targeted therapy can be investigated for specific types of sleep problems seen in hemodialysis patients. These investigations aim to provide a comprehensive understanding of sleep problems in hemodialysis patients, leading to the development of innovative methods that can enhance their quality of life and facilitate faster recovery.
